# Prehospital anesthesia in postcardiac arrest patients: a multicenter retrospective cohort study

**DOI:** 10.1186/s40001-024-01864-x

**Published:** 2024-05-02

**Authors:** Gerrit Jansen, Eugen Latka, Michael Bernhard, Martin Deicke, Daniel Fischer, Annika Hoyer, Yacin Keller, André Kobiella, Bernd Strickmann, Lisa Marie Strototte, Karl-Christian Thies, Kai Johanning

**Affiliations:** 1 University Department of Anesthesiology, Intensive Care Medicine and Emergency Medicine, Johannes Wesling Klinikum Minden, Ruhr University Bochum, Hans-Nolte-Straße 1, 32429 Minden, Germany; 2 Medical School OWL, Bielefeld University, Universitätsstraße 25, 33615 Bielefeld, Germany; 3 Department of Medical and Emergency Services, Study Institute Westfalen-Lippe, Remterweg 44, 33617 Bielefeld, Germany; 4 Central Emergency Department, University Hospital of Düsseldorf, Moorenstrasse 5, 40225 Düsseldorf, Germany; 5 Emergency Medical Service, Countryside of Osnabrueck, Am Schölerberg 1, 49082 Osnabrueck, Germany; 6 Department of Anesthesiology and Operative Intensive Care Medicine, Hospital of Osnabrueck, Am Finkenhügel 1, 49076 Osnabrueck, Germany; 7 Emergency Medical Service, City and District of Lippe-Detmold, Röntgenstraße 18, 32756 Detmold, Germany; 8 Biostatistics and Medical Biometry, Medical School OWL, Bielefeld University, Universitätsstraße 25, 33615 Bielefeld, Germany; 9 Department of Public Order and Security, Fire and Disaster Control Office, Integrated Regional Control Centre, Scharfenberger Straße 47, 01139 Dresden, Germany; 10 Departement for Anesthesiology and Intensive Care Medicine, Emergency Medicine and Pain Therapy, Municipal Hospital Dresden - Friedrichstadt, Friedrichstraße 41, 01067 Dresden, Germany; 11 Emergency Medical Service, City and District of Guetersloh, Herzebrocker Strasse 140, 33324 Guetersloh, Germany; 12 Emergency Medical Service, City and District of Guetersloh, Herzebrocker Strasse 140, 33324 Guetersloh, Germany; 13 Department of Anesthesiology, Intensive Care, Emergency Medicine, Transfusion Medicine, and Pain Therapy, Protestant Hospital of the Bethel Foundation, Medical School OWL, Bielefeld University, Burgsteig 13, 33617 Bielefeld, Germany; 14 Department of Anesthesiology, Operative Intensive Care Medicine, Emergency Medicine and Pain Therapy, Bielefeld Municipal Hospital, Medical School OWL, Bielefeld University, Campus Klinikum Bielefeld, Teutoburger Straße 50, 33604 Bielefeld, Germany

**Keywords:** CPR, Emergency medicine, Resuscitation, Postarrest care, Management

## Abstract

**Background:**

Currently, the data regarding the impact of prehospital postcardiac arrest anesthesia on target hemodynamic and ventilatory parameters of early postresuscitation care and recommendations on its implementation are rare. The present study examines the incidence and impact of prehospital postcardiac arrest anesthesia on hemodynamic and ventilatory target parameters of postresuscitation care.

**Methods:**

In this multicentre observational study between 2019 and 2021 unconscious adult patients after out-of-hospital-cardiac arrest with the presence of a return-of-spontaneous circulation until hospital admission were included. Primary endpoint was the application of postarrest anesthesia. Secondary endpoints included the medication group used, predisposing factors to its implementation, and its influence on achieving target parameters of postresuscitation care (systolic blood pressure: ≥ 100 mmHg, etCO_2_:35–45 mmHg, SpO_2_: 94–98%) at hospital handover.

**Results:**

During the study period 2,335 out-of-hospital resuscitations out of 391,305 prehospital emergency operations (incidence: 0.58%; 95% CI 0.54–0.63) were observed with a return of spontaneous circulation to hospital admission in 706 patients (30.7%; 95% CI 28.8–32.6; female: 34.3%; age:68.3 ± 14.2 years). Postcardiac arrest anesthesia was performed in 482 patients (68.3%; 95% CI 64.7–71.7) with application of hypnotics in 93.4% (*n* = 451), analgesics in 53.7% (*n* = 259) and relaxants in 45.6% (*n* = 220). Factors influencing postcardiac arrest sedation were emergency care by an anesthetist (odds ratio: 2.10; 95% CI 1.34–3.30; *P* < 0.001) and treatment-free interval ≤ 5 min (odds ratio: 1.59; 95% CI 1.01–2.49; *P =* 0.04). Although there was no evidence of the impact of performing postcardiac arrest anesthesia on achieving a systolic blood pressure ≥ 100 mmHg at the end of operation (odds ratio: 1.14; 95% CI 0.78–1.68; *P =* 0.48), patients with postcardiac arrest anesthesia were significantly more likely to achieve the recommended ventilation (odds ratio: 1.59; 95% CI 1.06–2.40; *P =* 0.02) and oxygenation (odds ratio:1.56; 95% CI 1.04–2.35; *P =* 0.03) targets. Comparing the substance groups, the use of hypnotics significantly more often enabled the target values for etCO2 to be reached alone (odds ratio:2.79; 95% CI 1.04–7.50; *P =* 0.04) as well as in combination with a systolic blood pressure ≥ 100 mmHg (odds ratio:4.42; 95% CI 1.03–19.01; *P =* 0.04).

**Conclusions:**

Postcardiac arrest anesthesia in out-of-hospital cardiac arrest is associated with early achievement of respiratory target parameters in prehospital postresuscitation care without evidence of more frequent hemodynamic complications.

**Supplementary Information:**

The online version contains supplementary material available at 10.1186/s40001-024-01864-x.

## Background

Out-of-hospital-cardiac arrest continues to be of extraordinary social relevance due to its high mortality and morbidity [1, 2]. In addition to strategies to shorten the treatment-free interval and to support early defibrillation, the importance of early, optimal postresuscitation care has received growing attention in recent years [3–5].

For prehospital emergency care in this context, early action according to the ABC concept; advanced airway management by means of intubation of the trachea performed by the most experienced provider available, in patients with persistent coma or other clinical indication for anesthesia; their capnographic control; sufficient ventilation with titration of FiO2 to achieve a reliably measured pulsoxymetric oxygen saturation (SpO2) between 94 and 98% while avoiding hyper- and hypoxemia and a target endtidal CO2 (etCO2) between 35 and 45 mmHg; the avoidance of hypotensive phases (target mean arterial pressure ≥ 65 mmHg respectively systolic blood pressure ≥ 100 mmHg) and targeted temperature management with temperatures between 32 and 36 °C is recommended [4, 6]. Based on these recommendations, both intubation of the trachea, if not already done during resuscitation, and optimal synchronization of the patient to the emergency respirator in this early phase of postresuscitation care, make postcardiac arrest anesthesia appear reasonable. In this context, various factors must be taken into account when weighing the pros and cons of postcardiac arrest anesthesia: Possible advantages are the reduction of oxygen consumption; facilitation of endotracheal intubation and improvement of tube tolerance as well as increased compliance with mechanical ventilation; induction of retrograde amnesia, analgesia and stress reduction; therapy of posthypoxic seizures; facilitated performance of interventions necessary immediately after hospital admission (e.g. coronary angiography); rapid induction of a possibly indicated target temperature management as well as potentially neuroprotective effects of various anesthetics [4, 7–11, 13–19]. Factors that count against postcardiac arrest anesthesia include hemodynamic side effects with possible re-arrest, acute hypotension and subsequentially reduced cerebral perfusion; the risk of unnecessary induction of target temperature management with therapy-associated complications; delayed awakening in the intensive care unit with the prolongation of the duration of ventilation and intensive care stay and more difficult prognosis assessment [4, 8, 10, 18]. To date, little data and no well founded recommendations for performing prehospital postcardiac arrest anesthesia exist [8–11]. The present multicenter observational study examines the frequency, performance, complications and impact of prehospital postcardiac arrest anesthesia on established target parameters of postresuscitation care in prehospital emergency medicine in Germany [4].

## Methods

The study was approved by the Institutional Review Board of the University of Muenster on 03.10.2022, Germany file reference 2022–617-f-S). Procedures were followed in accordance with the ethical standards of the responsible committee on human experimentation and with the Helsinki Declaration. Owing to its retrospective nature, the requirement of written informed consent was waived by the institutional review board. This article adheres to the applicable Strengthening the Reporting of Observational studies in Epidemiology (STROBE) guidelines.

The study was based on the electronically recorded rescue service data across three prehospital emergency medical services (city of Dresden, districts of Gütersloh and Lippe, Germany) with a total of approximately 1,275,000 inhabitants in the Federal Republic of Germany between 01.01.2019 and 31.12.2021. There were no differences between these centers with regards to the emergency physician who intubated and the preference for a particular anesthetic agent.

In Germany, in life-threating emergencies like out-of-hospital-cardiac arrest, a paramedic ambulance and a medical intervention car, staffed by a paramedic and an emergency physician, are dispatched to the Scene of the emergency.

In Germany, emergency physicians complete a 40-h course to participate in prehospital emergency medicine after at least 1.5 years of training in a specialty such as anesthesia, intensive care medicine or emergency care.

The respective patient care reports of the resuscitations performed were evaluated, supplemented by data from the German Resuscitation Register.

Patients ≥ 18 years of age with prehospital resuscitation for out-of-hospital cardiac arrest of any cause (e.g. cardiac, hypoxic, etc. [see Table 1]), defined according to the Utstein criteria as the need for chest compressions and/or defibrillation, unconsciousness after prehospital return-of-spontaneous circulation, and spontaneous circulation at hospital admission were included [12].

**Table 1:** Characteristics of the patients included, the emergency physicians involved and the resuscitation measures performed

Parameters	Overall [*n* = 706] [No. (%)]	Postarrest Anesthesia [*n* = 482 (68.3)] [No. (%)]	No Postarrest Anesthesia [*n* = 224 (31.7)] [No. (%)]
Age (years) [mean ± SD]	68.3 ± 14.2	68.6 ± 14.1	67.9 ± 14.3
Female Sex	242 (34.3)	166 (34.4)	76 (33.9)
Health status before the onset of circulatory arrest			
Pre-existing conditions without restrictions on everyday life	280 (39.7)	200 (41.5)	80 (35.7)
Pre-existing conditions with restrictions on everyday life	285 (40.4)	186 (38.6)	99 (44.2)
Normal daily life impossible	52 (7.4)	37 (7.7)	15 (6.7)
Missing data	89 (12.6)	59 (12.2)	30 (13.4)
Comorbidities			
Lungs	99 (14.0)	65 (13.5)	34 (15.2)
Cardiac	301 (42.6)	210 (43.6)	91 (40.6)
Neurologic	74 (10.5)	48 (9.9)	26 (11.6)
Metabolic	108 (15.3)	79 (16.4)	29 (12.9)
Malignancy	32 (4.5)	23 (4.8)	9 (4.0)
Immunodeficiency	4 (0.6)	(0.4)	2 (0.9)
Unknown/no data	232 (32.9)	96 (19.9)	136 (60.7)
Training level of emergency physicians			
Specialist	387 (54.8)	285 (59.1)	102 (45.5)
Physician in training	128 (18.1)	87 (18.0)	41 (18.3)
No data	191 (27.0)	110 (22.9)	81 (36.2)
Speciality			
Anesthesiology	354 (50.1)	260 (53.9)	94 (42.0)
Internal medicine	106 (15.0)	70 (14.5)	36 (16.1)
Surgery	46 (6.5)	38 (7.9)	8 (3.6)
Others	24 (3.4)	13 (3.9)	11 (4.9)
Unknown/no data	176 (24.9)	101 (20.9)	75 (33.5)
Presumed cause of cardiac arrest			
Cardiac	456 (64.6)	323 (67.0)	133 (59.4)
Hypoxia	138 (19.5)	93 (13.2)	45 (20.1)
Trauma	20 (2.8)	11 (2.3)	9 (4.0)
Metabolic	12 (1.7)	6 (1.2)	6 (2.7)
Hemorrhage to death	9 (1.3)	6 (1.2)	3 (1.3)
Others	9 (1.3)	6 (1.2)	3 (1.3)
Intoxication	7 (1.0)	4 (0.8)	3 (1.3)
Intracranial pathology	5 (0.7)	4 (0.8)	1 (0.4)
Sepsis	3 (0.4)	1 (0.2)	2 (0.9)
Unknown	47 (6.6)	28 (5.8)	19 (8.5)
Treatment-free interval			
< 5 Min	264 (37.4)	193 (40.0)	71 (31.7)
5–10 Min	82 (11.6)	56 (11.6)	26 (11.6)
> 10 Min	85 (12.1)	52 (10.8)	33 (14.8)
Unknown	275 (38.9)	181 (37.5)	94 (42.0)
Initial shockable rhythm	246 (34.8)	196 (40.7)	50 (22.3)
Number of defibrillations during resuscitation [mean ± SD]	2.1 ± 1.1	2.2 ± 1.2	2.0 ± 1.1
Initial airway management			
Extraglottic airway	207 (29.3)	129 (26.8)	78 (34.8)
Intubation of the trachea prior to ROSC	138 (19.5)	104 (21.6)	34 (15.2)
Intubation of the trachea after ROSC	122 (17.3)	72 (14.9)	50 (22.3)
Time of first epinephrine application			
Cumulative dose of epinephrine (mg) [mean ± SD]	3.5 ± 9.2	3.2 ± 8.6	4.3 ± 10.3
Duration of resuscitation (min †:sec §) [mean ± SD]	19:43 s ± 14:30	18:21 ± 14:26	22:31 ± 14:13
Complications in the course of the operation	345 (48.9)	237 (49.2)	108 (48.2)
Airway complications	132 (18.7)	99 (20.5)	33 (14.7)
Aspiration	18 (2.5)	10 (2.1)	8 (3.6)
Re-arrest	111 (15.7)	63 (13.1)	48 (21.4)
Hypotension	321 (45.5)	220 (45.6)	102 (45.5)
Antihypotensive therapy following cardiac arrest	318 (45.0)	220 (45.6)	98 (43.7)
Theodrenaline/Cafedrin (Akrinor®)	98 (13.9)	77 (16.0)	21 (9.4)
Norepinephrine	85 (12.0)	62 (12.9)	23 (10.3)
Epinephrine	196 (27.8)	113 (23.4)	83 (37.0)
Vital signs at handover			
SpO_2_			
< 94%	322 (45.6)	200 (41.5)	122 (54.5)
94–98%	194 (27.5)	146 (30.3)	48 (21.4)
> 98%	118 (16.7)	93 (19.3)	25 (11.2)
Missing	72 (10.2)	43 (8.9)	29 (12.9)
etCO_2_			
< 35 mmHg	323 (45.7)	211 (43.8)	112 (50.0)
35–45 mmHg	185 (26.2)	139 (28.8)	46 (20.5)
> 45 mmHg	110 (15.6)	76 (15.8)	34 (15.2)
Missing	88 (12.5)	56 (11.6)	32 (14.3)
SBP			
≥ 100 mmHg	396 (56.1)	281 (58.3)	115 (51.3)
Missing	250 (35.4)	166 (34.4)	84 (37.5)

EtCO_2_,  Endtidal CO_2_ concentration; min, minutes; SBP, systolic blood pressure; SD, standard deviation; Sec, seconds; SpO_2_,  peripheral Saturation of Oxygen; ROSC,  Return of spontaneous circulation

Exclusion criteria were no out-of-hospital-cardiac arrest, no resuscitative measures performed, patient age < 18 years; no occurrence of return-of-spontaneous circulation; consciousness after occurrence of return-of-spontaneous circulation; patients in whom out-of-hospital-cardiac arrest occurred as a result of prehospital sedation and/or induction of anesthesia; death findings; do-not-resuscitate order; no hospital admission; no presence of sustained spontaneous circulation on hospital admission; and incomplete data.

In addition to patient factors (age, sex, state of health before the onset of circulatory arrest, relevant comorbidities [cardiac, pulmonary, neurological, metabolic, malignant, immunodeficiency, not known]), the qualifications (doctor in training, specialist, not known) and specialization of the attending emergency physician (anesthesia, surgery, internal medicine, pediatrics, other, not known) were recorded.

In addition, data on circulatory arrest (suspected cause [cardiac, hypoxia, hemorrhage to death, trauma, sepsis, intracranial pathology, intoxication, metabolic, drowning, others, unknown]; duration of the treatment-free interval, defined as time between collapse and start of chest compressions [≤ 5 min, 5–10 min, > 10 min, unknown]; initial heart rhythm [shockable vs. nonshockable]; number of defibrillations performed, initial airway management [extraglottic airway, intubation of the trachea, intubation of the trachea after return-of-spontaneous-circulation]; time of first epinephrine application and cumulative dose of epinephrine (mg); duration of resuscitation], complications in the course of the operation (airway complications, difficult airway management, defined as > 1 intubation attempt or need for procedure change, aspiration, re-arrest, hypotension), the antihypotensive therapy used following cardiac arrest (theodrenaline/cafedrine, norepinephrine, epinephrine), the use of postarrest anesthesia, and vital signs at hospital transfer (SpO2, etCO2, systolic blood pressure) and grouped according to the recommended target parameters of postarrest treatment (SpO_2_ < 94%; 94–98%; > 98%; etCO_2_ < 35 mmHg, 35–45 mmHg, > 45 mmHg, systolic blood pressure ≥ 100 mmHg) [4].

The postarrest hemodynamic and ventilatory management was set according to the judgment of the attending physician. The data on prehospital postcardiac arrest anesthesia included the substance used (analgesics such as opioids, hypnotics, neuromuscular blocking agents), dose and any combinations of different drugs used.

The primary endpoint was the use of anesthetics [hypnotics and/or analgesics and/or neuromuscular blocking agents] after out-of-hospital-cardiac arrest and return-of-spontaneous-circulation until hospital admission. Secondary endpoints included training level and specialty of attending emergency physicians; factors associated with cardiac arrest (presumed cause of cardiac arrest, duration of treatment-free interval, duration of resuscitation; resuscitation measures performed); complications in the course of the operation; and achievement of guideline target parameters for oxygenation, ventilation and blood pressure.

### Statistical analysis

The data analysis and statistical plan was written and recorded in the investigators' files before data were accessed. We first performed a logistic regression to assess the association between specific covariates and allocation to postcardiac arrest anesthesia. As covariates we used the characteristics of the patients included, the emergency physicians involved and the resuscitation measures performed as shown in Table 1. In as second step we checked for potential association between allocation to postcardiac arrest anesthesia or nonpost cardiac arrest anesthesia and reaching the specific clinically relevant parameters of etCO2 within and without 35–45 mmHg, SpO2 within or without 94–98% and systolic blood pressure < 100 or ≥ 100 mmHg while adjusting for potential confounders using a logistic regression model. Odds ratio (OR) estimates are given with their 95% confidence intervals (95% CI) and p values. All analyses were performed using the statistical software SAS 9.4 (SAS Institute Inc, Cary, NC).

## Results

In the study period between 01.01.2019 and 31.12.2021, a total of 2,335 out-of-hospital resuscitations out of 391,305 emergency cases were recorded in the participating study centers (incidence of out-of-hospital resuscitation: 0.59% of prehospital emergency operations per year; 95% CI 0.57–0.62; approximately 183.1 per 100,000 inhabitants and year; 95% CI 175.8–190.7).

**Fig. 1:**
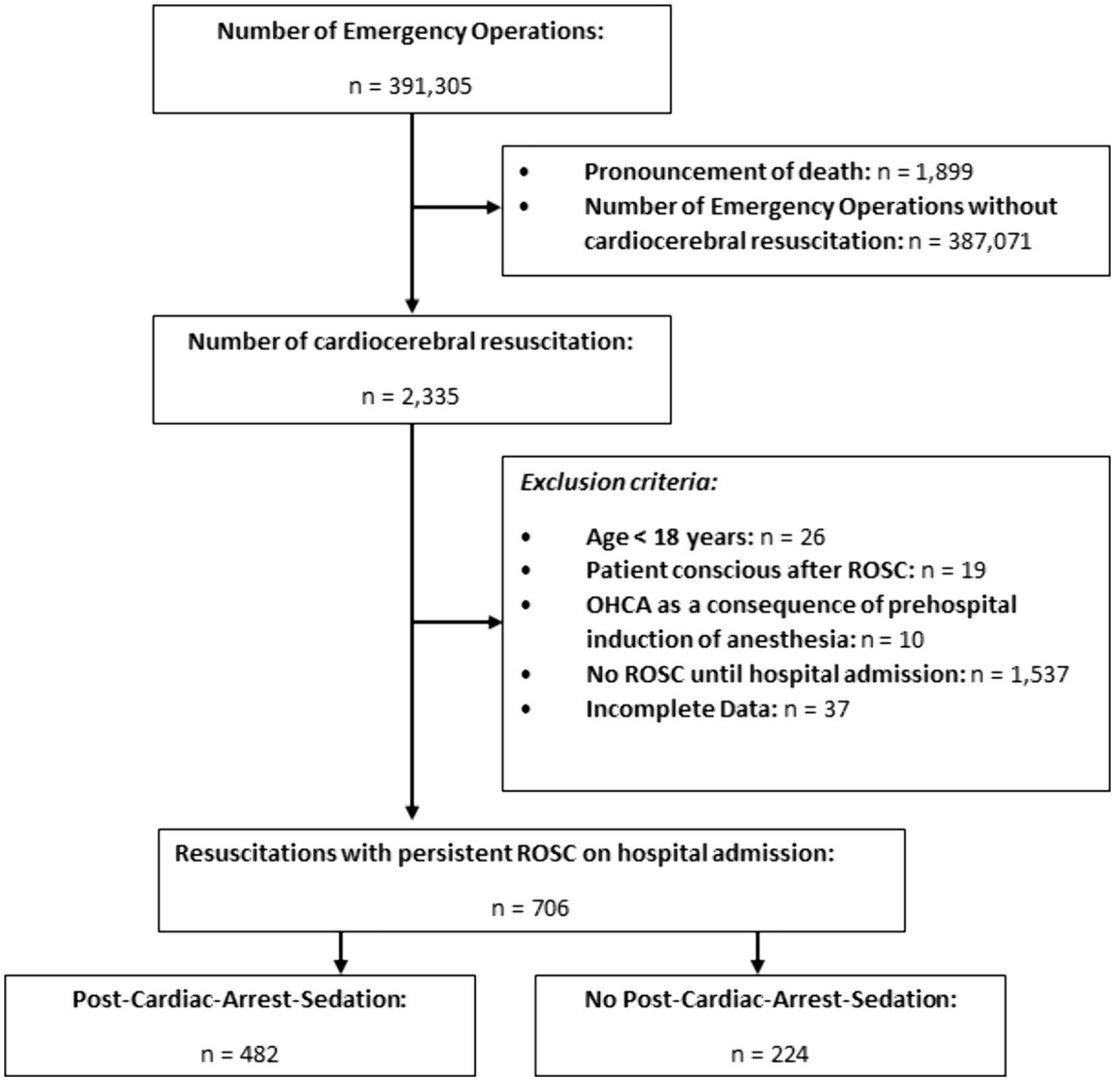
Flowchart of the applied inclusion and exclusion criteria. OHCA, out-of-hospital cardiac arrest; ROSC, return of spontaneous

Figure 1 shows an overview of the evaluated prehospital emergency operations after application of all inclusion and exclusion criteria. 2298 reports were included, while 37 were excluded with incomplete datasets.

In 706 (30.7%; 95% CI 28.8–32.6) of these patients, a stable return-of-spontaneous circulation was achieved at hospital admission. Table 1 shows the patient’s characteristics, the emergency physicians involved, the resuscitation measures performed, complications during the course of the operation and vital signs at the end of the operation.

**Fig. 2:**
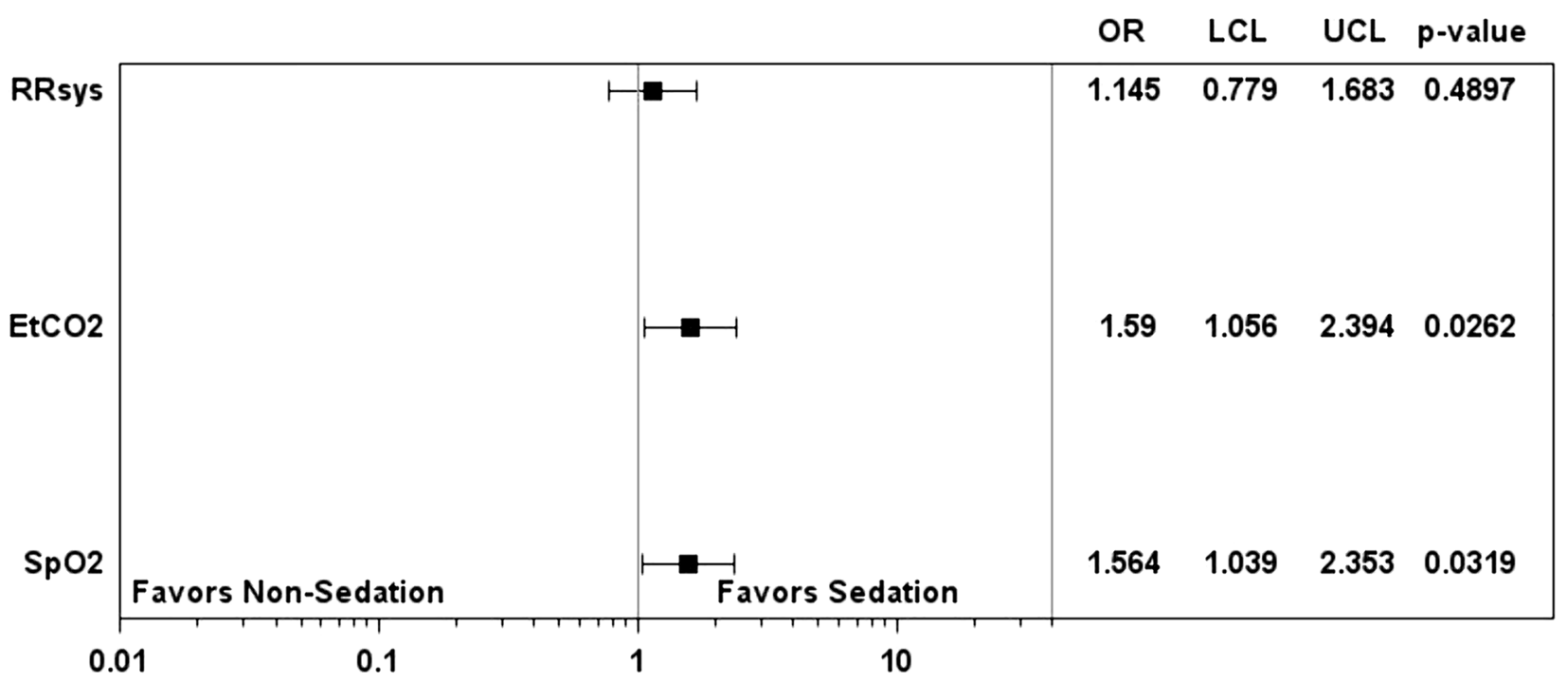
Odds ratios of target parameters of postrescuscitation care in comparison of the groups: postcardiac arrest anesthesia vs. non postcardiac arrest anesthesia. etCO_2_, endtidal CO_2_; LCL, lower confidence limit; OR, odds ratio; RRsys, systolic blood pressure; SpO_2_, peripheral oxygen saturation; UCL, upper confidence level

Postcardiac arrest anesthesia was performed in 482 (68.3%; 95% CI 64.7–71.7) patients. Table 2 shows an overview of the substances used. In 152 (31.5%; 95% CI 27.4–35.9) patients, postcardiac arrest anesthesia was performed for transition from an extraglottic airway device to endotracheal intubation. Table 3 shows the OR of the factors influencing the performance of postcardiac arrest anesthesia. Figure 2 shows the OR of the comparison of the two study groups regarding target parameters of postresuscitation therapy to be achieved at hospital admission (etCO_2_ 35–45 mmHg, SpO_2_ 94–98% and systolic blood pressure ≥ 100 mmHg). With regards to the achievement of oxygenation goals, there were no significant differences for hypoxic cause of arrest (OR: 1.34, 95% CI 0.76–2.57, *P =* 0.28); initial extraglottic airway (OR: 0.85, 95% CI 0.58–1. 25, *P* = 0.40); initial intubation of the trachea (OR: 0.91, 95% CI 0.53–1.55) nor for complications in securing the airway (OR: 1.54, 95% CI 0.97–2.44, *P* = 0.07). There were also no significant differences for achievement of ventilation goals when considering hypoxic cause of arrest (OR: 0.73, 95% CI 0.38–1.41, *P* = 0.35); initial extraglottic airway (OR: 1.09, 95% CI 0.75–1.60, *P* = 0.64); initial intubation of the trachea (OR: 1.448, 95% CI 0.81–2.60; complications in securing the airway (OR: 0.87, 95% CI 0.54–1.41, *P =* 0.57). There was no evidence for the influence of postcardiac arrest anesthesia on achieving a systolic blood pressure ≥ 100 mmHg (OR: 1.14, 95% CI 0.78–1.68, *P =* 0.49); antihypotensive therapy following out-of-hospital-cardiac arrest (OR: 0.55, 95% CI 0.28–1.06, *P =* 0.07); complications in the course of the operation (OR: 0.59; 95% CI 0.31–1.12, *P =* 0.10). A detailed overview of the OR of potential factors influencing the target parameters of the postresuscitation care is shown in Additional file 1. Additional file 2 presents an overview of the OR of the different anesthetics and their influence on the corresponding target parameters of postresuscitation management. The administration of hypnotics increased the probability of achieving an etCO2 35–45 mmHg (OR: 2.79; 95% CI 1.04–7.50; *P =* 0.04) as well as for jointly achieving an etCO2 35–45 mmHg in combination with a systolic blood pressure ≥ 100 mmHg (OR: 4.42; 95% CI 1.03–19.01; *P =* 0.04). Additional file 3 shows the comparison of midazolam and propofol and their influence on the target parameters of postresuscitation therapy. No significant differences were observed.

**Table 2:** Characteristics of the conducted postcardiac arrest anesthesia (*n* = 482)

	Overall (*n* = 482) [No. (%)]
Application of analgesics	259 (53.7)
Analgesic applied	
Fentanyl	237 (91.5)
Cumulative dose in mg [mean ± SD]	0.22 ± 0.14
Morphine	29 (12.2)
Cumulative dose in mg [mean ± SD]	6.5 ± 4.6
Application of hypnotics	450 (93.4)
Hypnotic applied	
Midazolam	381 (84.7)
Cumulative dose in mg [mean ± SD]	10.4 ± 5.8
Single shot-propofol	131 (29.1)
Average bolus-dose in mg [mean ± SD]	142.4 ± 93.0
Continuous infusion of Propofol	10 (2.2)
(S-)-Ketamine	25 (5.5)
Cumulative dose in mg [mean ± SD]	67.4 ± 50.7
Thiopental	1 (0.2)
Cumulative dose in mg [mean ± SD]	500 ± 0
Application of relaxant	220 (45.6)
Relaxant applied	
Rocuronium	164 (74.5)
Cumulative dose in mg [mean ± SD]	55.1 ± 17.5
Succinylcholine	42 (19,1)
Cumulative dose in mg [mean ± SD]	97.3 ± 30.1
Cis-atracurium	33 (15.0)
Cumulative dose in mg [mean ± SD]	11.1 ± 4.6
Combination of drugs	
Solely Analgesic	14 (2.9)
Solely Hypnotic	140 (29.0)
Solely Relaxant	2 (0.4)
Analgesic + Hypnotic	104 (21.6)
Analgesic + Relaxant	11 (2.3)
Hypnotic + Relaxant	77 (16.0)
Analgesic + Hypnotic + Relaxant	130 (27.0)

SD, standard deviation

**Table 3:** Overview of odds ratios comparing the groups: postcardiac arrest anesthesia vs. no-postarrest anesthesia

	Odds ratio	95% CI	*p* value
Age	0.99	0.98–1.01	0.61
Sex (Male vs. Female)	0.95	0.59–1.54	0.85
Pre-emergency status (pre-existing conditions with vs. without restrictions on everyday life)	0.84	0.52–1.36	0.48
Cardiac Cause of Arrest (Yes vs. no)	1.58	0.99–2.54	0.06
Anesthesiologist (Yes vs. no)	2.10	1.34–3.30	0.001
Treatment-free interval (≤ 5 min vs. > 5 min)	1.59	1.01–2.49	0.04
Complications in the course of the operation (Yes vs. no)	1.06	0.68–1.66	0.80

## Discussion

In this multicenter observational study prehospital postcardiac arrest anesthesia was performed in 68.3%. Factors associated with the performance of postcardiac arrest anesthesia were emergency medical care by an anesthesiologist or a treatment-free interval of ≤ 5 min. Patients who received postcardiac arrest anesthesia were significantly more likely to achieve the oxygenation and ventilation goals of postresuscitation care at hospital admission without evidence of an increased rate of hemodynamic complications.

Advanced airway management, as well as induction and maintenance of anesthesia represent high-risk procedures, especially in critically ill patients [8, 10, 13, 14]. To improve the quality and safety of care, recommendations for the management of intubation of the trachea and induction of anesthesia in critically ill and emergency patients have been developed [8, 10, 15–17]. In out-of-hospital cardiac arrest patients, intubation of the trachea is often performed during or soon after resuscitation. Induction of general anesthesia is sometimes useful for its implementation [4, 6, 15]. Surprisingly, the postcardiac arrest patient population has not been considered in the current recommendations and it has so far been insufficiently evaluated, whether these recommendations are directly transferable to postcardiac arrest patients [4, 8, 10, 16, 17]. The present study shows that prehospital postcardiac arrest anesthesia is frequently used and indicates the need to develop evidence-based recommendations and guidelines for its implementation.

The decision to initiate general anesthesia onsite is contingent upon several factors, including the feasibility within the operational setting (“stay and play” versus “load and go”). This entails considerations such as proximity to the nearest suitable hospital, available equipment (including potential spatial constraints within the emergency vehicle), the qualifications of the emergency medical personnel. In addition, the determination of whether to induce general anesthesia onsite is influenced by the confidence of the attending physician in administering such anesthesia. In this context, postcardiac arrest anesthesia was performed more frequently by anesthetists possibly due to their greater experience in anesthesia induction. The indications for anesthetic drugs could vary tremendously, and a binary definition may have influenced the biological significance of a dose–response relationship. However, based on the present findings, this would underline the importance of developing recommendations for postcardiac arrest anesthesia for nonanesthesiologists or for use in a nonphysician-based ambulance system.

The favorable influence of the shortest possible no-flow or low-flow times on prognosis is known from studies [5, 20, 21]. The higher likelihood of obtaining a postcardiac arrest anesthesia in the patient population with a treatment-free interval and therefore no-flow or low-flow times ≤ 5 min may indicate that these patients may possibly have shown signs of a more favorable outcome which made a postcardiac arrest anesthesia necessary (e.g. spontaneous respiration, return of protective reflexes) [21]. In particular, this subpopulation could benefit most from performing a postcardiac arrest anesthesia: In the postresuscitation phase, patients are at risk of hypoxic–ischemic and hyperoxemic–reperfusion brain injury [4, 22–24]. Control of paCO2 is important because of its importance for cerebral vascular tone besides avoidance of hypotensive phases and achievement of an adequate blood pressure, acting as determinant of cerebral perfusion [4]. The available data indicates that the implementation of prehospital postcardiac arrest anesthesia is associated with a significant improvement in the achievement of ventilation and oxygenation goals of postresuscitation care without evidence of an increased rate of hemodynamic complications or negative effects on the achievement of the recommended blood pressure goals. As part of a structured prehospital postresuscitation care, it could thus potentially be suitable to improve patient care in this particularly vulnerable phase, following the return-of-spontaneous circulation.

So far, there are no recommendations regarding the optimum medication for postcardiac arrest anesthesia [4]. The effects of different combinations of substances on intubation conditions and hemodynamics in critically ill patients have been investigated in numerous studies [8, 10, 16, 17, 25]. Midazolam and propofol are potent vasodilators, carrying the risk of circulatory depression when administered in usual induction doses. However, these are widely used in the induction of anesthesia in critically ill patients, as also shown in the present study [10, 11, 26]. The use of propofol is widespread in anesthesia, intensive care and emergency medicine, which may explain its frequent use in the present work. While the effects on hemodynamics during induction of anesthesia in critically ill patients are inconsistent in studies [10, 11, 16, 27], experimental data in animal models show that propofol in the context of postresuscitation care could potentially reduce cerebral oxygen consumption as well as ischemia–reperfusion injury resulting in better survival and neurological outcome in a mouse model [19, 28]. Ketamine was recommended in the hemodynamically unstable patient. However, in rapid sequence induction, ketamine was associated with cardiac arrests and even worse rates of hypotension compared to etomidate, which, despite its potentially beneficial hemodynamic effects, has long been controversial because of concerns about adrenal suppression and was not used in the present work [8, 10, 29, 30] However, the optimal drug combination for postcardiac arrest anesthesia is still unknown. The present study indicates that future studies are needed to detect the influence of postcardiac arrest anesthesia on survival and neurological outcome as well as an optimal substance or combination of substances within the framework of future structured prehospital postresuscitation care.

## Limitations

Limitations include the restrictions akin to retrospective studies e.g. underreporting. There is risk of selection bias, as patients who received anesthetic drugs and died in the prehospital setting were not included in the study. The proportion of patients with Out-of-hospital-cardiac arrest as a result of induction of anesthesia in the study cohort was low (Fig. 1). Confounding by indication cannot be excluded as it is likely that unconscious patients were not sedated. Possibly, patients who recover well from cardiac arrest and are then relatively hemodynamically stable are more likely to receive analgesic and hypnotic medications in comparison to their less stable counterparts. There are several unmeasured confounders that can impact the evaluated target parameters of postresuscitation care (e.g. ventilatory settings after intubation, use of positive end-expiratory pressure etc.). Furthermore, they refer to the handover of the patient in hospital and do not allow any statement concerning the time required to correct possible deviations from the recommended parameters during the operation. Due to technical limitations of prehospital monitoring equipment, such as SpO2 or noninvasive blood pressure in centralized or hypothermic patients, flawed measurements are possible. It is therefore possible that the proportion of patients who achieved the target parameters was underestimated. The etCO2 may be influenced by things such as aspiration of blood or gastric contents, cardiac output, or severity of lung injury and other reasons and therefore does not always correlate well with the arterial pCO2.

Furthermore, the present study investigated the effects of postcardiac arrest anesthesia on target parameters of hemodynamics, oxygenation and ventilation of postresuscitation care. Therefore, it does not allow any conclusions about the impact of postcardiac arrest anesthesia on patient outcome. However, the positive influence of achieving these target parameters on outcome has been proven in studies that form the basis of current guidelines [4.

## Conclusion

Prehospital postcardiac arrest anesthesia is frequent and predominantly executed using hypnotics. While prehospital postcardiac arrest anesthesia was associated with a significant improvement in the achievement of ventilation and oxygenation goals in the prehospital setting, the present study showed no evidence of an increased rate of hemodynamic complications or deviations from hemodynamic target parameters during transfer to hospital.

## Meetings

Parts of the present work have been presented as an abstract at the Congress of the German interdisciplinary association for intensive care and emergency medicines (DIVI 2022) 30.11.-02.12.2022, Hamburg, Germany and the German interdisciplinary emergency medicine congress (DINK 2023) 09.03.2023–10.03.2023, Koblenz, Germany.

### Supplementary Information

**Additional file 1.** Overview of odds ratios for target parameters of postresuscitation care.

**Additional file 2. **Overview of the odds ratios of the comparison of the different anesthetic groups.

**Additional file 3. **Comparison of the effects of midazolam vs. propofol.

## Data Availability

The datasets used and/or analysed during the current study are available from the corresponding author on reasonable request.

## Abbreviations

95% CI: 95% Confidence Intervall
etCO_2_: End-tidal CO_2_ concentration
OR: Odds ratio
SpO_2_: Pulse-oximetric oxygen saturation
