# Supplementary material for: Prehospital anesthesia in postcardiac arrest patients: a multicenter retrospective cohort study
Source: Eur J Med Res. 2024 May 2;29:263. doi: 10.1186/s40001-024-01864-x (PMC11067130; doi:10.1186/s40001-024-01864-x)
Supplement: Supplementary file 1 — Additional file 1. Overview of odds ratios for target parameters of postresuscitation care. [file 40001_2024_1864_MOESM1_ESM.docx]

**Additional file 1:** Overview of odds ratios for target parameters of postresuscitation care

| **Parameters of postresuscitation care** | **Odds Ratio** | **CI 95%** | **p value** |
| --- | --- | --- | --- |
| **SpO2 94-98 %** |  |  |  |
| **Postcardiac arrest anesthesia (Yes vs. no)** | ***1.56*** | ***1.04 – 2.35*** | ***0.03*** |
| Age | 0.99 | 0.98-1.01 | 0.64 |
| Sex (Male vs. Female) | 0.96 | 0.66 – 1.41 | 0.84 |
| Cardiac Cause of Arrest (Yes vs. no) | 0.83 | 0.49 – 1.34 | 0.48 |
| Antihypotensive therapy following cardiac arrest (Yes vs. no) | 0.68 | 0.34-1.34 | 0.26 |
| Complications in the course of the operation (Yes vs. no) | 1.04 | 0.53-2.03 | 0.91 |
| Hypoxic Cause of Arrest (Yes vs. no) | 1.40 | 0.76 – 2.57 | 0.28 |
| Initial Supraglottic Airway (Yes vs. no) | 0.85 | 0.58 – 1.25 | 0.40 |
| Initial Endotracheal Intubation (Yes vs. no) | 0.91 | 0.53 – 1.55 | 0.72 |
| Complications in securing the airway (Yes vs. no) | 1.54 | 0.97 – 2.44 | 0.07 |
| ***EtCO2 35-45 mmHg*** |  |  |  |
| **Postcardiac arrest anesthesia (Yes vs. no)** | ***1.59*** | ***1.06-2.40*** | ***0.02*** |
| Age | 1.00 | 0.99-1.02 | 0.45 |
| Sex (Male vs. Female) | 0.83 | 0.56-1.22 | 0.34 |
| Cardiac cause of arrest (Yes vs. no) | 1.05 | 0.61-1.79 | 0.87 |
| Antihypotensive therapy following cardiac arrest (Yes vs. no) | 1.71 | 0.83-3.52 | 0.15 |
| Complications in the course of the operation (Yes vs. no) | 0.63 | 0.31-1.30 | 0.21 |
| Hypoxic cause of arrest (Yes vs. no) | 0.73 | 0.38 – 1.41 | 0.35 |
| Initial supraglottic airway (Yes vs. no) | 1.09 | 0.75 – 1.60 | 0.64 |
| Initial Endotracheal Intubation (Yes vs. no) | 1.45 | 0.81 – 2.60 | 0.21 |
| Complications in securing the airway (Yes vs. no) | 0.87 | 0.54 – 1.41 | 0.57 |
| **Reaching blood pressure target SBP ≥ 100 mmHg** |  |  |  |
| Postcardiac arrest anesthesia (Yes vs. no) | 1.14 | 0.78 – 1.68 | 0.49 |
| Age | 0.99 | 0.98 – 1.00 | 0.08 |
| Sex (Male vs. Female) | 0.95 | 0.65 – 1.39 | 0.80 |
| Cardiac cause of arrest (Yes vs. no) | 0.99 | 0.66 – 1.48 | 0.96 |
| Antihypotensive therapy following cardiac arrest (Yes vs. no) | 1.54 | 0.79 – 2.99 | 0.20 |
| Complications in the course of the operation (Yes vs. no) | 0.55 | 0.28 – 1.06 | 0.07 |
| Pre-emergency status (pre-existing conditions with vs. without restrictions on everyday life) | 1.12 | 0.77 – 1.64 | 0.54 |
| Shockable vs. nonshockable initial rhythm | 1.45 | 0.94 – 2.23 | 0.09 |
| Dosis of Epinephrine | 0.99 | 0.95 – 1.03 | 0.65 |
| **Reaching blood pressure target SBP ≥ 100 mmHg ant etCO2 35-45 mmHg** |  |  |  |
| Postcardiac arrest anesthesia (Yes vs. no) | 1.55 | 0.98-2.46 | 0.06 |
| Age | 0.99 | 0.98-1.01 | 0.81 |
| Sex (Male vs. Female) | 0.82 | 0.53-1.25 | 0.35 |
| Cardiac cause of arrest (Yes vs. no) | 1.32 | 0.85-2.06 | 0.21 |
| Antihypotensive therapy following cardiac arrest (Yes vs. no) | 1.18 | 0.54-2.57 | 0.67 |
| Complications in the course of the operation (Yes vs. no) | 0.86 | 0.40-1.88 | 0.71 |
| Legend:  EtCO2 = endtidal CO2; SpO2 = peripheral Oxygen saturation; SBP =systolic blood pressure | | | |
