# Supplementary material for: Prehospital anesthesia in postcardiac arrest patients: a multicenter retrospective cohort study
Source: Eur J Med Res. 2024 May 2;29:263. doi: 10.1186/s40001-024-01864-x (PMC11067130; doi:10.1186/s40001-024-01864-x)
Supplement: Supplementary file 2 — Additional file 2. Overview of the odds ratios of the comparison of the different anesthetic groups. [file 40001_2024_1864_MOESM2_ESM.docx]

**Additional file 2:** Overview of the odds ratios of the comparison of the different anesthetic groups

|  | **Odds Ratio** | **CI 95%** | **p value** |
| --- | --- | --- | --- |
| **SBP ≥ 100 mmHg** |  |  |  |
| ***Age*** | ***0.98*** | ***0.97-1.00*** | ***0.04*** |
| Sex (Male vs. Female) | 1.11 | 0.73-1.68 | 0.63 |
| Analgesics (Yes vs. no) | 0.75 | 0.50-1.12 | 0.16 |
| Hypnotics (Yes vs. no) | 1.45 | 0.69-3.06 | 0.33 |
| Relaxant (Yes vs. no) | 1.08 | 0.73-1.62 | 0.69 |
| **SpO2** **94-98%** |  |  |  |
| Age | 1.00 | 0.99-1.02 | 0.69 |
| Sex (Male vs. Female) | 1.20 | 0.77-1.85 | 0.42 |
| Analgesics (Yes vs. no) | 1.13 | 0.75-1.70 | 0.57 |
| Hypnotics (Yes vs. no) | 1.41 | 0.60-3.20 | 0.43 |
| Relaxant (Yes vs. no) | 0.97 | 0.64-1.46 | 0.87 |
| **etCO2 35-45 mmHg** |  |  |  |
| Age | 1.00 | 0.98-1.02 | 0.97 |
| Sex (Male vs. Female) | 0.74 | 0.47-1.15 | 0.17 |
| Analgesics (Yes vs. no) | 1.14 | 0.75-1.74 | 0.54 |
| **Hypnotics (Yes vs. no)** | ***2.79*** | ***1.04-7.50*** | ***0.04*** |
| Relaxant (Yes vs. no) | 1.40 | 0.92-2.13 | 0.12 |
| **SBP ≥ 100 mmHg + etCO2 35-45 mmHg** |  |  |  |
| Age | 0.99 | 0.98-1.01 | 0.38 |
| Sex (Male vs. Female) | 0.77 | 0.47-1.27 | 0.31 |
| Analgesics (Yes vs. no) | 1.10 | 0.68-1.77 | 0.71 |
| ***Hypnotics (Yes vs. no)*** | ***4.42*** | ***1.03-19.01*** | ***0.04*** |
| Relaxant (Yes vs. no) | 1.38 | 0.86-2.22 | 0.18 |
| Legend:  EtCO2 = endtidal CO2; SpO2 = peripheral Oxygen saturation; SBP =systolic blood pressure | | | |
