# Supplementary material for: Prehospital anesthesia in postcardiac arrest patients: a multicenter retrospective cohort study
Source: Eur J Med Res. 2024 May 2;29:263. doi: 10.1186/s40001-024-01864-x (PMC11067130; doi:10.1186/s40001-024-01864-x)
Supplement: Supplementary file 3 — Additional file 3. Comparison of the effects of midazolam vs. propofol. [file 40001_2024_1864_MOESM3_ESM.docx]

**Additional file 3:** Comparison of the effects of midazolam vs. propofol

|  | **Odds Ratio** | **CI 95%** | **p value** |
| --- | --- | --- | --- |
| **SBP ≥ 100 mmHg** |  |  |  |
| Age | 0.98 | 0.97-0.99 | 0.02 |
| Sex (Male vs. Female) | 1.09 | 0.72-1.65 | 0.68 |
| Midazolam (Yes vs. no) | 0.78 | 0.45 – 1.35 | 0.37 |
| Propofol (Yes vs. no) | 0.61 | 0.37 – 1.01 | 0.05 |
| **SpO2 94-98%** |  |  |  |
| Age | 1.00 | 0.99 - 1.02 | 0.60 |
| Sex (Male vs. Female) | 1.20 | 0.78 - 1.86 | 0.40 |
| Midazolam (Yes vs. no) | 1.65 | 0.92 – 2.98 | 0.09 |
| Propofol (Yes vs. no) | 1.19 | 0.71 – 2.01 | 0.5120 |
|  |  |  |  |
| **etCO2 35-45 mmHg** |  |  |  |
| Age | 0.99 | 0.98 - 1.01 | 0.94 |
| Sex (Male vs. Female) | 0.76 | 0.49 - 1.17 | 0.22 |
| Midazolam (Yes vs. no) | 1.18 | 0.66 – 2.11 | 0.57 |
| Propofol (Yes vs. no) | 1.00 | 0.58 – 1.71 | 0.99 |
|  |  |  |  |
| **SBP ≥ 100 mmHg + etCO2 35-45 mmHg** |  |  |  |
| Age | 0.99 | 0.97 - 1.01 | 0.31 |
| Sex (Male vs. Female) | 0.80 | 0.49 - 1.31 | 0.36 |
| Midazolam (Yes vs. no) | 0.93 | 0.48 – 1.76 | 0.81 |
| Propofol (Yes vs. no) | 1.04 | 0.57 – 1.91 | 0.89 |
| Legend: EtCO2 = endtidal CO2; SpO2 = peripheral Oxygen saturation; SBP =systolic blood pressure | | | |
